# Toxicity and outcomes of thoracic re-irradiation using stereotactic body radiation therapy (SBRT)

**DOI:** 10.1186/1748-717X-8-99

**Published:** 2013-04-25

**Authors:** Marsha Reyngold, Abraham J Wu, Amanda McLane, Zhigang Zhang, Meier Hsu, Nicholas F Stein, Ying Zhou, Alice Y Ho, Kenneth E Rosenzweig, Ellen D Yorke, Andreas Rimner

**Affiliations:** 1 Department of Radiation Oncology, Memorial Sloan-Kettering, 1275 York Ave, New York, NY, 10065, USA; 2 Epidemiology and Biostatistics, Memorial Sloan-Kettering, New York, NY, USA; 3 Medical Physics, Memorial Sloan-Kettering, New York, NY, USA; 4 Radiation Oncology, Mount Sinai Medical Center, New York, NY, USA

**Keywords:** Lung re-irradiation, SBRT, Pulmonary toxicity, BED

## Abstract

**Background:**

Patients treated for a thoracic malignancy carry a significant risk of developing other lung lesions. Locoregional control of intrathoracic recurrences is challenging due to the impact of prior therapies on normal tissues. We examined the safety and efficacy of thoracic re-irradiation using high-precision image-guided stereotactic body radiation therapy (SBRT).

**Methods:**

Records of 39 patients with prior intra-thoracic conventionally fractionated radiation therapy (RT) who underwent SBRT for a subsequent primary, recurrent or metastatic lung tumor from 11/2004 to 7/2011 were retrospectively reviewed.

**Results:**

Median dose of prior RT was 61 Gy (range 30–80 Gy). Median biologically effective prescription dose (α/β = 10) (BED_10_) of SBRT was 70.4 Gy (range 42.6-180 Gy). With a median followup of 12.6 months among survivors, 1- and 2-year actuarial local progression-free survival (LPFS) were 77% and 64%, respectively. Median recurrence-free (RFS) and overall survival (OS) were 13.8 and 22.0 months, respectively. Patients without overlap of high-dose regions of the primary and re-irradiation plans were more likely to receive a BED_10_ ≥100 Gy, which was associated with higher LPFS (hazard ratio, [HR] = 0.18, p = 0.04), RFS ([HR] = 0.31, p = 0.038) and OS ([HR] = 0.25, p = 0.014). Grade 2 and 3 pulmonary toxicity was observed in 18% and 5% of patients, respectively. Other grade 2–4 toxicities included chest wall pain in 18%, fatigue in 15% and skin toxicity in 5%. No grade 5 events occurred.

**Conclusions:**

SBRT can be safely and successfully administered to patients with prior thoracic RT. Dose reduction for cases with direct overlap of successive radiation fields results in acceptable re-treatment toxicity profile.

## Background

Thoracic re-irradiation is not uncommonly encountered in the clinic as a considerable number of patients with primary or metastatic lung lesions require repeat treatments to the thorax for recurrent or metachronous disease. Lung cancer remains the second most common malignancy in the U.S. with an estimated 221,130 new cases diagnosed in 2011, and as many as 61% of all lung cancer patients receive radiotherapy (RT) at initial presentation [1]. Although the majority of relapses are systemic, a considerable percentage is limited to the thorax, which represents a subset of patients who may benefit from local therapy. Early-stage patients have a 4–10% chance of developing a metachronous second lung tumor in the first 5 years after treatment [2-4]. Patients with stage III non-small cell lung cancer (NSCLC) treated with concurrent chemoradiation have a 25% risk of isolated locoregional failure within the radiation field [5]. In addition, the lungs are a common site for metastases from other malignancies, where local therapy to the thorax may be indicated for palliation of symptoms.

Re-irradiation of the thorax for recurrent lung cancer with conventionally fractionated RT has yielded 1-year local progression-free (LPFS) and overall survival (OS) of up to 51% and 59%, respectively [6-10]. Image-guided hypofractionated stereotactic body radiation therapy (SBRT) is a useful modality for repeat treatments because it allows for delivery of high radiation dose to the target while minimizing the dose to the surrounding tissue. SBRT has been shown to be highly effective in the treatment of small lung lesions including early-stage lung cancer [11,12] as well as pulmonary metastasis [13-15]. A number of different fractionation schemes have been used. Their relative biological effectiveness can be approximated using a widely accepted formula of biologically equivalent dose (BED), where the alpha beta ratio (α/β) is a radiosensitivity parameter unique to each tumor tissue and outcome, and is commonly set to 10 for lung tumor control and acute toxicity. SBRT fractionation schemes with BED for α/β = 10 (BED_10_) ≥100 Gy were associated with improved outcomes [11]. However, the efficacy and risk of SBRT in the setting of re-irradiation is not well characterized. Kelly et al. recently described their experience with SBRT after prior conventionally fractionated RT in 36 patients [16]. They predominantly employed 50 Gy in 4 fractions and reported excellent 2-year local control (LC), progression-free survival (PFS) and OS rates of 95%, 26% and 59% respectively, although at the expense of a 64% rate of grade 2–3 pneumonitis. This series was subsequently updated by Liu et al. with a larger number of patients focusing on identifying risk factors for radiation pneumonitis [17]. In a smaller series employing a number of different fractionation schemes, Trakul et al. described inferior outcomes in 15 patients with 1-year local control of 65.5%, but with minimal toxicity [18]. Here we review our institutional experience using SBRT in 39 patients who had received prior fractionated RT with emphasis on predictive factors for clinical outcomes and toxicity.

## Methods

After obtaining IRB approval for retrospective review of data in our institutional database, we identified all patients with a prior history of conventionally fractionated RT to the thorax who underwent hypofractionated re-irradiation with SBRT at Memorial Sloan-Kettering Cancer Center (MSKCC) from 11/2004 to 7/2011 (Table 1). Patients with prior RT to the breast were excluded. Thirty-nine patients met the inclusion criteria and their records were reviewed for patient and disease characteristics, prior fractionated RT dosimetric variables, SBRT dosimetric variables, length of time from the end of SBRT to local and regional relapse, length of survival, and toxicity.

**Table 1:** Patient characteristics at the time of SBRT

**Patient characteristics at the time of SBRT**	
Median age, years (range)	71 (41–94)
Median KPS (range)	80 (60–100)
Gender, N	
Female	19
Male	20
History of tobacco use, N (%)	39 (100%)
COPD, N (%)	22 (56%)
Median follow-up, months (range)	12.6 (1.3-47.5)
Median interval between fractionated RT and SBRT, months (range)	37 (1–180)
Type of initial radiation, N	
Conventional	13
3D-CRT	12
IMRT	14
Median prior radiation dose, Gy (range)	61 (30–80)
History of thoracic surgery, N (%)	21 (54%)
History of chemotherapy, N (%)	22 (56%)
Diagnosis, N	
Primary lung cancer	17
Stage I-II	15
Stage III	1
Stage IV	1
Recurrent lung cancer	18
Localized	16
Metastatic	2
Other histologies	4

*KPS,* Karnofsky performance scale; *3D-CRT*, 3D-Conformal radiation therapy; *IMRT*, Intensity modulated radiation therapy.

Previous RT variables reviewed included dates of therapy, prescription dose, graphic dose distributions, planning target volume (PTV) size, location and coverage, mean lung doses and V20 for the ipsilateral and total lungs. Complete previous RT records were available for 22 patients who received either three-dimensional conformal radiotherapy (3D-CRT) or intensity-modulated radiotherapy (IMRT) at MSKCC (n = 21) or an outside institution (n = 1). Seven patients treated at MSKCC and 2 at outside institutions received two-dimensional treatments, typically AP-PA and off-cord boost fields. To obtain dosimetric information for these 9 patients, their plans were reconstructed using prior portal images and current computed tomography (CT) scans.

For SBRT planning, a four-dimensional (4D) CT simulation scan was performed with the patient immobilized in a custom-designed Alpha Cradle® device. The internal target volume was delineated by combining gross tumor volume projections from each breathing phase of the 4D scan. A 2-3 mm expansion was used to create a clinical target volume, and a 5 mm expansion was used to create a PTV. SBRT treatment plans were generated using an in-house treatment planning system [19,20] to deliver the prescribed dose to the PTV using IMRT. The dose was prescribed to the isodose line covering the PTV (generally 100% isodose line). Patients were treated using multi-field coplanar beam arrangements typically consisting of 4–6 beams. The degree of overlap between the previous conventionally fractionated treatment and SBRT was evaluated by review of beam films and by image registration between old and new planning scans. Risk-adapted SBRT fractionation schemes were based on PTV size and degree of overlap between areas from conventionally fractionated RT and SBRT (Table 2). Prescription BED_10_ ≤ 60 Gy was more frequently considered for cases with PTV volumes > 70 cc and/or complete overlap. SBRT dosimetric parameters analyzed included PTV size, coverage, mean dose to ipsilateral lung, mean dose to both lungs, volume receiving 20 Gy (V20) of ipsilateral lung and V20 of both lungs. Mean lung doses were calculated based on the total lung volume minus GTV. To estimate composite mean lung doses from fractionated RT and SBRT, the SBRT mean dose was converted to 2 Gy equivalents using the linear-quadratic model (α/β = 3 Gy) and added to the total mean dose of the first course of fractionated RT.

**Table 2:** SBRT characteristics

**SBRT Characteristics**	**Total**	**Overlap with prior RT**	**No overlap**
	**N = 39**	**N = 22**	**N = 17**
Median BED_10_ , Gy (range)	70 (43–180)	48 (43–106)	106 (58–180)
BED_10_ ≥ 100 Gy, N (%)	15 (38)	4 (18)	11 (65)
60 Gy in 3 fr (BED_10_ = 180 Gy)	3		
54 Gy in 3 fr ( BED_10_ = 151 Gy)	4		
48 Gy in 4 fr ( BED_10_ = 106 Gy)	8		
BED_10_ < 100 Gy, N (%)	24 (62)	18 (82)	6 (35)
40-45 Gy in 5 fr ( BED_10_ = 72-86 Gy)	4		
20-22 Gy in 1 fr ( BED_10_ = 60-70 Gy)	4		
32-35 Gy in 4–5 fr ( BED_10_ = 54-59 Gy)	4		
27.5-30 Gy in 5 fr ( BED_10_ = 43-48 Gy)	12		
Median PTV size, cm^3^ (range)	67 (17–473)	117 (20–473)	45 (17–197)
Median GTV size, cm^3^ (range)	19 (0.7-227)	43 (2–227)	6 (0.7-74)
Median ipsilateral lung mean dose, Gy (range)	6.3 (1.7-13.1)	4.8 (1.7-13.1)	6.6 (1.7-13.1)
Median combined lung mean dose, Gy (range)	3.2 (0.8-7.2)	3.1 (1.1-6.2)	4.3 (0.8-7.2)
Median ipsilateral lung V20, % (range)	10 (2–30)	10 (2–30)	12 (3–25)
Median combined lung V20, % (range)	5 (1–12)	5 (1–10)	5 (2–12)

*BED*_*10*_, Biologically equivalent dose for α/β = 10; *PTV*, Planning treatment volume; *GTV,* Gross tumor volume; *V20*, Percent of volume receiving 20 Gy or more.

Patients were clinically evaluated approximately 1 month after completion of SBRT and every 3–6 months thereafter with clinical exams and chest CT scans.

The primary endpoints were local progression-free survival (LPFS) and toxicity. LPFS was defined as the time from the end of SBRT until the date of local progression or last clinic visit if the patient did not progress. Local progression was defined as an enlarging mass in the SBRT treatment field and was confirmed by PET (6 of 10 cases with local progression) and biopsy (4 cases), if feasible. Pulmonary toxicity, including dyspnea, hypoxia, cough and pneumonitis, was graded according to CTCAE v4.0. For analysis of factors associated with pulmonary toxicity, a binary toxicity endpoint was defined as pulmonary toxicity within 6 months. Six of 39 patients in the study group who did not develop toxicity and had follow-up of less than 6 months were excluded. Secondary endpoints were recurrence-free survival (RFS) and overall survival (OS). Actuarial LPFS, RFS and OS from the date of SBRT were estimated with the Kaplan-Meier method. Cox regression models were used to evaluate prognostic factors for each of the endpoints. Cut points used for continuous variables in this analysis were based on clinical relevance (BED_10_) or dichotomized at the approximate median (time from conventionally fractionated RT to SBRT, KPS, and PTV size). Multivariable analysis was precluded by the small number of events. Fisher’s exact and Wilcoxon rank sum tests were used to evaluate the association between risk of toxicity and RT parameters.

## Results

### Patient and tumor characteristics

At the time of SBRT, the median patient age was 71 years (range 41–94 years) and the median Karnofsky Performance Score (KPS) was 80 (range 60–100) (Table 1). Twenty-two patients (56%) had documented chronic obstructive pulmonary disease. Seventeen patients received SBRT for a metachronous primary lung cancer, 18 for recurrent lung cancer, and 4 for metastases from other histologies. The criteria used for classifying tumors as a second primary rather than a recurrence included different histology, time interval between cancers of at least two years, and location within different lobes in the absence of tumor in the lymphatics [21].

### Prior RT characteristics

Thirty-eight patients received one prior course of fractionated RT to the thorax with a median prescription dose of 61 Gy (range, 30–80 Gy) (Table 2). One patient received two prior courses of fractionated RT (74 Gy and 56.7 Gy) to the ipsilateral lung. The indications for prior RT included definitive therapy for NSCLC (n = 30), SCLC (n = 6), and esophageal cancer (n = 2), adjuvant post-operative therapy for NSCLC (n = 4) and mesothelioma (n = 1), and palliation (n = 5). Fractionated RT was delivered using conventional two-dimensional technique (n = 13), 3D-CRT (n = 12), and IMRT (n = 14). The median interval between fractionated thoracic RT and SBRT was 37 months (range 1–180 months). Two patients had planned conventionally fractionated RT and SBRT within a short interval for oligometastatic disease and synchronous primaries (1 and 5 months, respectively).

### SBRT characteristics

A variety of risk-adapted SBRT fractionation schemes were used with a median BED_10_ of 70.4 Gy (range 42.6-180 Gy) (Table 2). Twenty-two patients had overlap of the high-dose region defined as ≥ 50% isodose lines (IDL) of the SBRT field with the prior fractionated RT field. SBRT prescription doses in patients with overlap between the treatment fields were lower than in patients without overlap (median BED_10_ = 48 Gy vs. 106 Gy). The median PTV treated with SBRT was 67 cc (range 17–473 cc). The median PTV for 22 patients with overlap was 117 cc (range 20–473 cc), while the median PTV of 17 patients with less or no overlap was 45 cc (range 17–197 cc). Fifteen patients (38%) received doses with prescription BED_10_ above 100 Gy. Of these, four had overlap of the high-dose region of the SBRT field with the prior conventionally fractionated RT field. Dosimetric parameters including V20 and mean lung doses for ipsilateral or both lungs are listed in Table 2.

### Local control and survival

**Figure 1:**
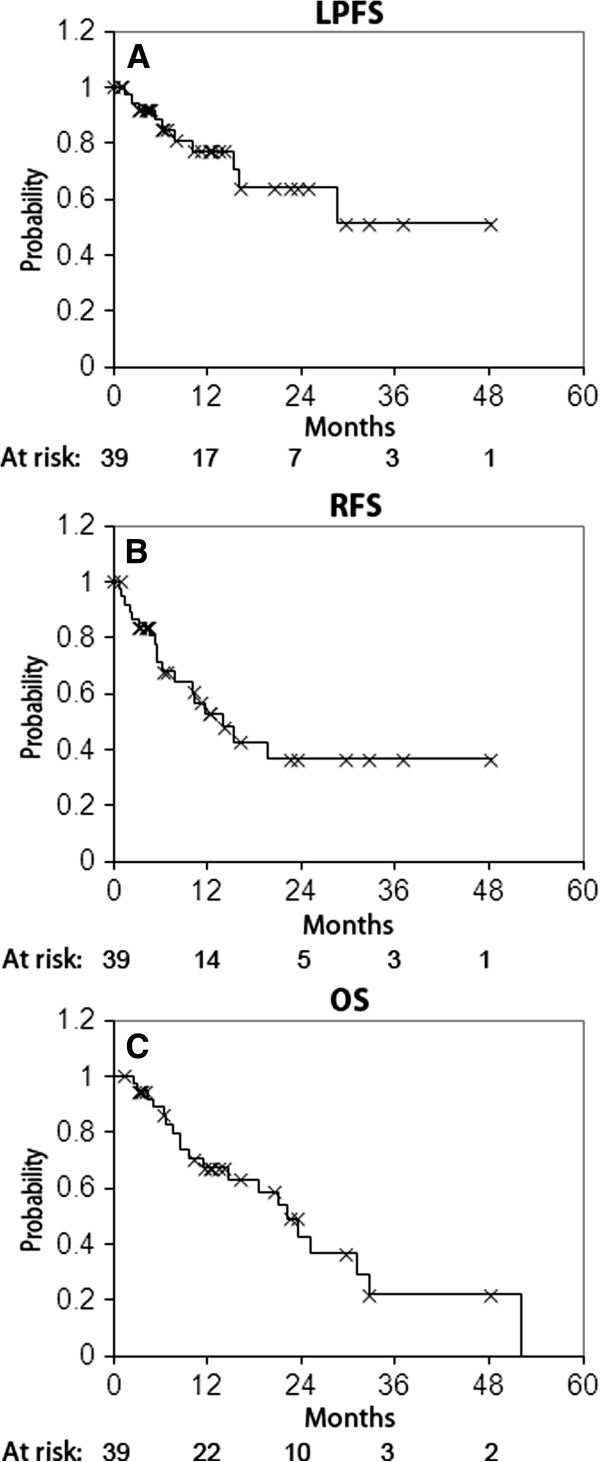
**Kaplan-Meier analyses of LPFS, RFS and OS.** LPFS (**A**), RFS (**B**) and OS (**C**) were calculated from the end of SBRT. Number at risk is displayed below each graph.

With a median follow up of 12.6 months (range 1.3-47.5 months), the actuarial LPFS was 77% at 1 year and 64% at 2 years (Figure 1). The actuarial median LPFS was not reached, whereas median RFS and OS were 13.8 and 22.0 months, respectively. On univariate analysis, no overlap with the prior RT field (hazard ratio [HR] = 0.11, p = 0.04), BED_10_ of ≥100 Gy (HR = 0.18, p = 0.04), time interval of > 36 months between conventionally fractionated RT and SBRT (HR = 0.25, p = 0.05), PTV < 75 cc (HR = 0.09, p = 0.03) and KPS ≥ 80 (HR = 0.16, p = 0.03) were associated with longer LPFS (Table 3). Having a metachronous primary lung cancer was borderline significant for a higher LPFS (p = 0.06).

**Table 3:** Factors associated with LPFS, RFS and OS on univariate analysis

	**LPFS**		**RFS**		**OS**	
**Characteristic**	**HR (95% CI)**	**p-value**	**HR (95% CI)**	**p-value**	**HR (95% CI)**	**p-value**
Type of Cancer						
Recurrent/metastasis	Reference		Reference		Reference	
Second Primary	0.26 (0.07, 1.04)	0.06	0.16 (0.05, 0.51)	0.002	0.37 (0.14, 0.99)	0.05
Overlap with prior RT field						
Yes	Reference		Reference		Reference	
No	0.11 ( 0.01,0.88)	0.04	0.52 (0.19, 1.39)	0.19	0.69 (0.27, 1.77)	0.44
BED_10_ of SBRT						
<100 Gy	Reference		Reference		Reference	
≥100 Gy	0.18 (0.04, 0.90)	0.04	0.31 (0.10, 0.93)	0.038	0.25 (0.08, 0.76)	0.014
Time from prior RT to SBRT						
≤36 months	Reference		Reference		Reference	
>36 months	0.25 (0.06,0.99)	0.05	0.37 (0.14, 0.97)	0.04	0.63 (0.25, 1.56)	0.32
PTV volume						
>75 cc	Reference		Reference		Reference	
16-75 cc	0.09 (0.01,0.79)	0.03	0.30 (0.10, 0.90)	0.03	0.27 (0.10, 0.71)	0.008
KPS						
<80	Reference		Reference		Reference	
≥80	0.16 (0.03, 0.85)	0.03	0.43 (0.10, 1.95)	0.28	0.11 (0.03, 0.36)	0.0003

*BED*_*10*_, Biologically equivalent dose for α/β = 10; *PTV*, Planning treatment volume; *KPS,* Karnofsky performance scale.

Similarly, longer RFS and OS were associated with the diagnosis of a second primary cancer (HR = 0.16, p = 0.002 and HR = 0.37, p = 0.05, respectively), BED_10_ of ≥100 Gy (HR = 0.31, p = 0.038 and HR = 0.25, p = 0.014, respectively), and PTV volume <75 cc (HR = 0.30, p = 0.03 and HR = 0.27, p = 0.008, respectively. In addition, KPS of ≥80 was associated with longer OS (HR = 0.11, p = 0.0003).

Multivariate analysis was precluded by the limited number of patients and events in the dataset.

### Toxicity

Grade 2 and 3 pulmonary toxicity, including dyspnea, hypoxia, cough and pneumonitis, were observed in 7 and 2 patients, respectively at a median of 3.0 months (range 1–6 months) (Table 4). Grade 2 pulmonary symptoms resolved in 2 of 6 patients for whom further follow-up was available, and continued to be reported by 4 patients, including 2 with poorly controlled COPD, 1 patient with hemoptysis due to disease progression, and 1 patient who developed a malignant pleural effusion shortly after undergoing SBRT. Patients who developed grade 3 toxicity continued to be oxygen-dependent at 23 and 6 months of follow-up. There was no clear correlation of any examined dosimetric variables with pulmonary toxicity. When patients with grade <2 and ≥2 pulmonary toxicity were compared, there were no statistically significant differences in V20 of prior fractionated RT or SBRT to the ipsilateral lung or total lungs. Similarly, there was no difference in mean dose, whether prior fractionated treatment and SBRT were considered separately, or as a composite of both treatments together (data not shown). SBRT doses with a BED_10_ ≥100 Gy vs. <100 Gy and overlap of prior conventionally fractionated RT field with SBRT field were also not predictive of pulmonary toxicity. Of note, 4 of 22 patients with overlap of conventionally fractionated RT field with SBRT field received doses with BED_10_ ≥ 100 Gy (48 Gy in 4 fractions). Only one of these patients developed grade 2 dyspnea, and none developed grade 3 or higher pulmonary events.

**Table 4:** Grade ≥ 2 toxicities (CTCAE v4.0)

**Grade ≥ 2 Toxicity (CTCAE 4.0)**	**N (%)**
Pulmonary (dyspnea, hypoxia, cough, pneumonitis)	9 (23%)
Grade 2	7
Grade 3	2
Chest wall pain	7 (18%)
Grade 2	5
Grade 3	2
Fatigue	6 (15%)
Grade 2	6
Grade 3	0
Skin/soft tissue	2 (5%)
Grade 2	1
Grade 3	0
Grade 4	1

Other grade 2 or higher toxicities included chest wall pain in 7 patients (18%), fatigue in 6 patients (15%) and skin/connective tissue toxicity in 2 patients (5%). There were no grade 2 or higher brachial plexopathy or esophageal toxicity. With the exception of one patient with grade 4 skin toxicity [22], no grade 4 or 5 events were observed.

## Discussion

Patients with a history of a primary lung cancer or pulmonary metastases are at a significant risk of developing other malignant lung lesions that may benefit from aggressive local treatment. Their therapeutic options are often limited, and RT represents an important modality in this setting. Image-guided hypofractionated stereotactic body radiation therapy (SBRT) is particularly useful and effective for small lung lesions.

Here we describe a cohort of patients with a history of conventionally fractionated thoracic RT who received SBRT using a variety of fractionation schemes. In the primary setting, LC rates for early-stage NSCLC treated with SBRT consistently exceed 90% [12,23-26]. Importantly, doses with a BED_10_ ≥100 Gy delivered to the isocenter have been reported to be associated with improved LC and survival [11]. Similarly, in the setting of previously un-irradiated lungs, SBRT for pulmonary metastases leads to high rates of LC up to 95- 96% at two years when high BED_10_ regimens are used [13,27]. However, in the setting of re-irradiation, concerns for increased toxicity frequently limit the radiation doses used with SBRT. Consistent with previously published single institution series [16-18], we show that acceptable rates of LC can be achieved with SBRT in this setting. One possible reason that our LC (2-year LC 64%) is lower than that reported by Kelly et al. (2-year LC 92%) may be related to our risk-adapted approach of selecting fractionation schemes with lower BED_10_ for patients with direct overlap between the high-dose regions of SBRT and the prior conventionally fractionated fields. Nevertheless, the local progression-free rate at 1 and 2 years in our study (77% and 64%, respectively) compare favorably with historical results of conventionally fractionated re-irradiation (51% and 42%, respectively) [6] or 1-year results of SBRT experience reported by Trakul et al. (65.5%). The OS reported here (median survival 22.0 months) is superior to that achieved with conventional re-irradiation in a similar group of 18 patients treated with definitive intent (median survival 15 months) [8] and is similar to that in other SBRT re-irradiation series (estimated median survival 21–26 months) [16,18].

Factors that would predict for a favorable outcome in the setting of re-irradiation are largely unknown, but may be clinically useful in selecting best candidates for re-irradiation or in deciding on the appropriate SBRT dose. We found that in select patients with previously irradiated lungs, doses of BED_10_ ≥100 Gy may be feasible and associated with good LPFS, RFS and OS without an unacceptably high risk of toxicity. In addition to BED_10_, other factors associated with improved LPFS on univariate analysis include receiving treatment for recurrent vs. metachronous primary lung cancer (typically early-stage), no overlap with prior RT, longer interval from prior RT to SBRT and smaller PTV volume. The type of lesion (recurrent vs. metachronous), overlap with prior RT, interval from prior RT to SBRT and PTV volume also correlate with RFS, whereas the type of lesion, PTV volume and KPS correlate with OS. Some of these factors are likely interrelated, and some may be more important than others in predicting outcomes, but multivariate analysis was precluded by the small number of events in this study. A larger cohort is needed to determine the relative importance of these potential prognostic factors.

Similar to the series reported by Trakul et al., we observed a low rate of grade 3 or higher pulmonary toxicity of 5% [18]. In contrast, the original and updated MDAnderson reports described a 28% and 20.8% incidence of grade 3 or higher radiation pneumonitis, respectively [16,17]. Some of these differences are likely related to differences in dose schedules used. While 72-100% of patients in the two MD Anderson series were treated with 50 Gy in 4 fractions (BED_10_ > 100 Gy), 62% of patients in our cohort received fractionation schemes with lower effective doses (BED_10_ < 100 Gy), chosen at the discretion of the treating physician, with the aim of limiting toxicity. In addition, the median interval between fractionated RT and SBRT was longer in our study (37 months vs. 21–22 months) which may, at least in part, contribute to the observed difference.

Liu et al. have identified a number of risk factors for severe pneumonitis, including ECOG status, FEV1, V20 of the composite plan and presence or absence of overlap with prior RT [17]. Despite evaluating a number of similar clinical and dosimetric parameters, we were unable to identify patient or treatment factors that may help predict which patients are at increased risk of developing toxicity in our cohort. This is likely related to the overall low number of toxic events. Taken together these studies suggest that for patients at very high risk for radiation pneumonitis according to the MD Anderson criteria, alternative fractionation schemes with lower BED_10_ may be considered. Further studies are necessary to better define the risk-benefit ratios of different fractionation schemes.

## Conclusion

SBRT can be used for re-treatment of previously irradiated lung. Consistent with data in the primary setting, fractionation schemes with BED_10_ ≥ 100 Gy appear to have more durable responses in the setting of re-irradiation on univariate analysis. Treatment toxicity may be reduced by using a risk-adapted approach with lower BED_10_ for lesions with direct overlap with the previously irradiated field. Further studies to address the risk-benefit ratios of different SBRT fractionation schemes in the context of previously irradiated lung are warranted.

## Consent

Waiver of HIPAA Authorization for the use of protected health information was obtained from the IRB (WA0132-07).

## Abbreviations

3D-CRT: 3D-Conformal radiation therapy; BED10: Biologically equivalent dose for α/β = 10; GTV: Gross tumor volume; KPS: Karnofsky performance scale; IMRT: Intensity modulated radiation therapy; LPFS: Local progression-free survival; OS: Overall survival; PTV: Planning treatment volume; RFS: Recurrence-free survival; SBRT: Stereotactic body radiation therapy; V20: Percent of volume receiving 20 Gy or more

## Competing interests

Kenneth E. Rosenzweig- Consultant role for chartrounds.com, Honoraria for lectures-ASTRO, ASCO, Expert testimony- Bookman, LLP.

Andreas Rimner – Consultant role for GE.

Other authors–none.

## Authors’ contributions

MR participated in study design, data acquisition, analysis and interpretation, and drafting of the manuscript; AJW and EDY made substantial contributions to study design, data interpretation and manuscription preparation; AM, NFS and YZ participated in data acquisition; ZZ and MH performed statistical analysis; AYH helped coordinate data acquisition; KER created the prospective SBRT database, helped coordinate data acquisition and contributed to data interpretation; AR conceived of the study, participated in the design, coordination, and data interpretation, and helped draft the manuscript. All authors contributed to manuscript revisions. All authors read and approved the final manuscript.

## References

[B1] Tyldesley S Boyd C Schulze K Walker H Mackillop WJ Estimating the need for radiotherapy for lung cancer: an evidence-based, epidemiologic approach Int J Radiat Oncol Biol Phys 2001 49 973 985 10.1016/S0360-3016(00)01401-2 11240238

[B2] Jeremic B Shibamoto Y Acimovic L Nikolic N Dagovic A Aleksandrovic J Radosavljevic-Asic G Second cancers occurring in patients with early stage non-small-cell lung cancer treated with chest radiation therapy alone J Clin Oncol 2001 19 1056 1063 11181669 10.1200/JCO.2001.19.4.1056

[B3] Martini N Bains MS Burt ME Zakowski MF McCormack P Rusch VW Ginsberg RJ Incidence of local recurrence and second primary tumors in resected stage I lung cancer J Thorac Cardiovasc Surg 1995 109 120 129 10.1016/S0022-5223(95)70427-2 7815787

[B4] Carr SR Schuchert MJ Pennathur A Wilson DO Siegfried JM Luketich JD Landreneau RJ Impact of tumor size on outcomes after anatomic lung resection for stage 1A non-small cell lung cancer based on the current staging system J Thorac Cardiovasc Surg 2012 143 390 397 10.1016/j.jtcvs.2011.10.023 22169444

[B5] Curran WJ Jr Paulus R Langer CJ Komaki R Lee JS Hauser S Movsas B Wasserman T Rosenthal SA Gore E Sequential vs. concurrent chemoradiation for stage III non-small cell lung cancer: randomized phase III trial RTOG 9410 J Natl Cancer Inst 2011 103 1452 1460 10.1093/jnci/djr325 21903745 PMC3186782

[B6] Wu KL Jiang GL Qian H Wang LJ Yang HJ Fu XL Zhao S Three-dimensional conformal radiotherapy for locoregionally recurrent lung carcinoma after external beam irradiation: a prospective phase I-II clinical trial Int J Radiat Oncol Biol Phys 2003 57 1345 1350 10.1016/S0360-3016(03)00768-5 14630272

[B7] Tada T Fukuda H Matsui K Hirashima T Hosono M Takada Y Inoue Y Non-small-cell lung cancer: reirradiation for loco-regional relapse previously treated with radiation therapy Int J Clin Oncol 2005 10 247 250 10.1007/s10147-005-0501-1 16136369

[B8] Okamoto Y Murakami M Yoden E Sasaki R Okuno Y Nakajima T Kuroda Y Reirradiation for locally recurrent lung cancer previously treated with radiation therapy Int J Radiat Oncol Biol Phys 2002 52 390 396 10.1016/S0360-3016(01)02644-X 11872284

[B9] Green N Melbye RW Lung cancer: retreatment of local recurrence after definitive irradiation Cancer 1982 49 865 868 10.1002/1097-0142(19820301)49:5<865::AID-CNCR2820490507>3.0.CO;2-H 7059924

[B10] Montebello JF Aron BS Manatunga AK Horvath JL Peyton FW The reirradiation of recurrent bronchogenic carcinoma with external beam irradiation Am J Clin Oncol 1993 16 482 488 10.1097/00000421-199312000-00004 8256761

[B11] Onishi H Araki T Shirato H Nagata Y Hiraoka M Gomi K Yamashita T Niibe Y Karasawa K Hayakawa K Stereotactic hypofractionated high-dose irradiation for stage I nonsmall cell lung carcinoma: clinical outcomes in 245 subjects in a Japanese multiinstitutional study Cancer 2004 101 1623 1631 10.1002/cncr.20539 15378503

[B12] Timmerman R Paulus R Galvin J Michalski J Straube W Bradley J Fakiris A Bezjak A Videtic G Johnstone D Stereotactic body radiation therapy for inoperable early stage lung cancer JAMA 2010 303 1070 1076 10.1001/jama.2010.261 20233825 PMC2907644

[B13] Rusthoven KE Kavanagh BD Burri SH Chen C Cardenes H Chidel MA Pugh TJ Kane M Gaspar LE Schefter TE Multi-institutional phase I/II trial of stereotactic body radiation therapy for lung metastases J Clin Oncol 2009 27 1579 1584 10.1200/JCO.2008.19.6386 19255320

[B14] Le QT Loo BW Ho A Cotrutz C Koong AC Wakelee H Kee ST Constantinescu D Whyte RI Donington J Results of a phase I dose-escalation study using single-fraction stereotactic radiotherapy for lung tumors J Thorac Oncol 2006 1 802 809 10.1097/01243894-200610000-00008 17409963

[B15] Ernst-Stecken A Lambrecht U Mueller R Sauer R Grabenbauer G Hypofractionated stereotactic radiotherapy for primary and secondary intrapulmonary tumors: first results of a phase I/II study Strahlenther Onkol 2006 182 696 702 10.1007/s00066-006-1577-x 17149575 PMC3233368

[B16] Kelly P Balter PA Rebueno N Sharp HJ Liao Z Komaki R Chang JY Stereotactic body radiation therapy for patients with lung cancer previously treated with thoracic radiation Int J Radiat Oncol Biol Phys 2010 78 1387 1393 10.1016/j.ijrobp.2009.09.070 20381271 PMC3401019

[B17] Liu H Zhang X Vinogradskiy YY Swisher SG Komaki R Chang JY Predicting radiation pneumonitis after stereotactic ablative radiation therapy in patients previously treated with conventional thoracic radiation therapy Int J Radiat Oncol Biol Phys 2012 84 1017 1023 10.1016/j.ijrobp.2012.02.020 22543216 PMC3612879

[B18] Trakul N Harris JP Le QT Hara WY Maxim PG Loo BW Jr Diehn M Stereotactic ablative radiotherapy for reirradiation of locally recurrent lung tumors J Thorac Oncol 2012 7 1462 1465 10.1097/JTO.0b013e31825f22ce 22895143

[B19] Mohan R Barest G Brewster LJ Chui CS Kutcher GJ Laughlin JS Fuks Z A comprehensive three-dimensional radiation treatment planning system Int J Radiat Oncol Biol Phys 1988 15 481 495 10.1016/S0360-3016(98)90033-5 3403328

[B20] Chui CS LoSasso T Spirou S Dose calculation for photon beams with intensity modulation generated by dynamic jaw or multileaf collimations Med Phys 1994 21 1237 1244 10.1118/1.597206 7799865

[B21] Martini N Melamed MR Multiple primary lung cancers J Thorac Cardiovasc Surg 1975 70 606 612 170482

[B22] Hoppe BS Laser B Kowalski AV Fontenla SC Pena-Greenberg E Yorke ED Lovelock DM Hunt MA Rosenzweig KE Acute skin toxicity following stereotactic body radiation therapy for stage I non-small-cell lung cancer: who’s at risk? Int J Radiat Oncol Biol Phys 2008 72 1283 1286 10.1016/j.ijrobp.2008.08.036 19028267

[B23] Fakiris AJ McGarry RC Yiannoutsos CT Papiez L Williams M Henderson MA Timmerman R Stereotactic body radiation therapy for early-stage non-small-cell lung carcinoma: four-year results of a prospective phase II study Int J Radiat Oncol Biol Phys 2009 75 677 682 10.1016/j.ijrobp.2008.11.042 19251380

[B24] Uematsu M Shioda A Suda A Fukui T Ozeki Y Hama Y Wong JR Kusano S Computed tomography-guided frameless stereotactic radiotherapy for stage I non-small cell lung cancer: a 5-year experience Int J Radiat Oncol Biol Phys 2001 51 666 670 10.1016/S0360-3016(01)01703-5 11597807

[B25] Nagata Y Takayama K Matsuo Y Norihisa Y Mizowaki T Sakamoto T Sakamoto M Mitsumori M Shibuya K Araki N Clinical outcomes of a phase I/II study of 48 Gy of stereotactic body radiotherapy in 4 fractions for primary lung cancer using a stereotactic body frame Int J Radiat Oncol Biol Phys 2005 63 1427 1431 10.1016/j.ijrobp.2005.05.034 16169670

[B26] Baumann P Nyman J Hoyer M Wennberg B Gagliardi G Lax I Drugge N Ekberg L Friesland S Johansson KA Outcome in a prospective phase II trial of medically inoperable stage I non-small-cell lung cancer patients treated with stereotactic body radiotherapy J Clin Oncol 2009 27 3290 3296 10.1200/JCO.2008.21.5681 19414667

[B27] Norihisa Y Nagata Y Takayama K Matsuo Y Sakamoto T Sakamoto M Mizowaki T Yano S Hiraoka M Stereotactic body radiotherapy for oligometastatic lung tumors Int J Radiat Oncol Biol Phys 2008 72 398 403 10.1016/j.ijrobp.2008.01.002 18374506

